# Net Platelet Angiogenic Activity (NPAA) Correlates with Progression and Prognosis of Non-Small Cell Lung Cancer

**DOI:** 10.1371/journal.pone.0096206

**Published:** 2014-04-30

**Authors:** Lijuan Yao, Hang Dong, Yiqin Luo, Jianping Du, Wen Hu

**Affiliations:** 1 Department of Laboratory Medicine, The Affiliated Anhui Provincial Hospital of Anhui Medical University, Hefei, People's Republic of China; 2 The Center of Tumor Therapy, The Affiliated Anhui Provincial Hospital of Anhui Medical University, Hefei, People's Republic of China; 3 Department of Pathology, The Affiliated Anhui Provincial Hospital of Anhui Medical University, Hefei, People's Republic of China; Ospedale Pediatrico Bambino Gesu', Italy

## Abstract

Circulating platelets are abundant sources of angiogensis molecules for the tumor vasculature affecting tumor growth and metastasis. The relationship between non-small cell lung cancer (NSCLC) and intra-platelet levels of VEGF, TSP-1 and net platelet angiogenic activity (NPAA) is unclear. The aim of this study was to better understand the role of these factors in the progression of NSCLC cancer and to assess its clinical significance. Platelet VEGF and TSP-1 and NPAA were measured preoperatively in 68 patients with NSCLC by ELISA or Capillary tube formation assay. VEGF, TSP-1 and NPAA distributions in cancer patients and healthy volunteers were compared using the Mann-Whitney *U* test. The Kaplan-Meier method, univariate and multivariate regression analysis was used to analyze the correlation between these factors and clinicopathological features, overall survival and disease-free survival. Mean intra-platelet TSP-1 level was slightly higher in patients than in healthy subjects (p = 0.092). Intra-platelet TSP-1 levels were significantly higher in patients with involvement greater than T2 or stage III, compared to other patients. Mean intra-platelet VEGF level was 40.8 pg/10^6^ in patients compared to 21.9 ng/10^6^ in healthy subjects (p = 0.041). Median value of NPAA in patients was significantly higher than that in healthy controls (p<0.001). Patients with high NPAA are more likely to exhibit aggressive clinical pathological features. NPAA greater than the median are associated with poor prognosis. The elevated NPAA have better correlation with tumor microvessel density (MVD) than platelet-derived VEGF. The areas under receiver operating curve (AUROC) of NPAA were higher than that of platelet derived VEGF in different groups. A multivariate analysis showed that NPAA are independent prognostic factors. These results indicated that NPAA may be a clinically useful indicator for diagnostic and prognostic evaluation in NSCLC patients.

## Introduction

Lung cancer is one of the most common malignancies worldwide, with a constantly increasing frequency, especially in China. It ranks as the first leading cause of cancer death among males in China [1], [2]. Despite advances in chemotherapy, prognosis for lung cancer patients remains poor, with 5-year relative survival less than 14% among males and less than 18% among females in most countries [3]. Thus, the ability to predict individual prognosis would be critical to guide surgical and chemotherapeutic treatment. Angiogenesis, or newblood vessel formation, an essential step in tumor growth and metastasis,is regulated by a balance between pro-angiogenic factors such as VEGF and bFGF, and anti-angiogenic factors such as endostatin and TSP-1. Platelets have long been known to be primary mediators of thrombosis and hemostasis. Recently, they have been shown to possess many angiogenesis promotors and angiogenesis inhibitors,being one of the largest single sources of angiogenic factors in vivo [4]. VEGF expression has been found to be high in various malignant tumors, and serum VEGF levels correlate with disease stage and prognosis in breast cancer, colorectal cancer and prostate cancer [5], [6]. It has been reported that high VEGF immunoreactivity in tumor tissues is associated with prognosis in NSCLC patients [7], [8]. Thrombospondin-1 (TSP-1) was the first endogenous inhibitor of angiogenesis to be identified [9], [10]. This 450-kDa glycoprotein is produced and secreted by several types of tumor cells and cells residing in the tumor stroma. The expression of TSP-1 in human tumors is modulated by oncogenes and microenvironmental factors, and in several kinds of human tumors TSP-1 is present in both the stroma and tumor cells [11]. Moreover, elevated circulating levels of TSP-1 have been associated with disease progression and advanced clinical stages of the disease [12], [13]. Although serum VEGF and TSP-1 are the most studied pro- and antiangiogenic factors, respectively, and highly elevated in angiogenesis-related diseases, studies regarding platelet-derived VEGF and TSP-1 are scarce in lung cancer patients. Recent studies demonstrated that, in addition to release of pre-existing compounds, platelets can synthesize new ones upon stimulation due to a large amount of messenger RNA [14]. This fact indicates that platelets may play a previously unknown role in regulating vascular responses. Herein, we have investigated VEGF and TSP-1, but also net platelet angiogenic activity (NPAA) in the platelets of lung cancer patients as compared to healthy controls. To evaluate the NPAA, we tested the in vitro effect of platelet lysates from patients with lung cancer on the formation of human umbilical cord endothelial cells (HUVECs) capillary-like structures.

## Materials and Methods

### Cell isolation and culture

Human umbilical cord endothelial cells (HUVECs) were obtained from umbilical cords provided by the local Department of Obstetrics. Written informed consent was obtained from the donors, and the study was approved by Ethics Committee of Anhui Medical University. Isolation and culturing was performed as described previously [15]. Cells were grown in RPMI medium supplemented with FBS (10%), L-glutamine (2 mM), streptomycin (100 µg/ml) and penicillin (100 U/ml) at 37°C in a humidified 5% CO_2_ incubator.

### Capillary tube formation assay

The tube formation assay was carried out according to the manufacturer's instructions (Chemicon International, Hampshire, UK) with slight modifications. Endothelial cells (1×10^4^) were seeded in growth factor-reduced matrigel coated plates (Becton Dickinson Biosciences, Bedford, MA, USA) and exposed to platelet lysates for 18 h. Tubule formation was examined under an inverted light microscope and the number of branch points in four non-overlapping fields was measured. Photographs of all wells were taken using an inverted light microscope. Experiments were performed in triplicate, and the variation was less than 10%. To evaluate the test reproducibility, capillary tube formation (CTF) of HUVEC were repeated 3 times in the presence of 9 selected patient platelets (3 with stimulatory activity, 3 with stimulatory activity in the range of healthy donor platelets, and 3 with inhibitory activity). In all of the repeats, the selected platelets maintained their original activity. To inhibit the activity of selected platelets, they were preimcubated with 5µg/ml VEGF monoclonal antibody (Sigma) or 200 µg/ml TSP antibody (Sigma).

### Patients and specimens

Sixty-eight patients with histologically or cytologically confirmed NSCLC at our hospital between September 2006 and July 2007 were included in the study. The 68 patients consisted of 46 men and 22 women (age 61±12.8 years [mean±SD]). The control group consisted of 68 healthy donors enrolled from the Blood Transfusion Unit of the same hospital during the same period. Both healthy donors and patients did not have a previous history of malignant disease. Clinical-pathological characteristics of patients were reported. Histologic classification of the tumors was based on the World Health Organization criteria [16]. Histologically, 37 tumors were adenocarcinoma, 28 were squamous cell carcinoma, and three were large cell carcinoma. Metastasis to the lymph nodes was N0 + N1 in 49 patients, N2+ N3 in 19. Postoperative disease stage was stage I + stage II in 38 patients, stage III + stage IV in 30. Paraffin-embedded, formalin-fixed surgical specimens were collected for immunohistochemical staining for intratumoral microvessel endothelial cells. The histopathology present in the archived frozen tissues was confirmed by a pathologist to be similar to that in the paraffin-embedded tissues. The survival time of patients was calculated from the date of operation to the date of death. The follow-up period lasted up to 60 months. Peripheral blood samples from patients with NSCLC were drawn within 1 week before surgery or chemotherapy. All blood samples from patients and controls were taken early in the morning, after an overnight fast and a rest period of at least 30 min. The samples were processed according to standard methods. Written informed consent was obtained from all participants, and the study protocols have been approved by Ethics Committee of Anhui Medical University.

### Preparation of human platelets

Standard “blue top” anticoagulation specimens were collected from each individual, and processed according to standard methods for platelet collection within 1–2 h of collection. Briefly, a blood sample (approximately 4 mL) was drawn by venipuncture into a vacuum tube containing 105 mM citrate (pH 5) anticoagulant at a ratio of 1∶9 (vol/vol) buffer to blood. The tubes were gently mixed and kept on a slow rocker at ambient temperature (20 to 28°C) while awaiting processing. Platelet rich plasma is obtained from citrated plasma by centrifugation (1000 rpm, 10 minutes) within 1 hour of collection, the volume recorded and a cell count performed. The platelet rich plasma is then centrifuged in the presence of PGI2 (75 nM) at 2000 rpm for 20 minutes to obtain a platelet pellet that is then washed twice in phosphate-buffered saline (PBS) and re-suspended gently in PBS to give a concentration of 2×10^8^ platelets/ml. one ml of this platelet suspension is mixed with 1 ml 0.1%Triton X-100 (Sigma) at 37°C for 20 minutes. This effectively lyses all the platelets. This platelet lysate is then stored at −80°C before analysis by ELISA.

### Measurements of VEGF and TSP-1

All of the samples were tested in the same laboratory, within the same timeframe (2 weeks). The cancer and control group samples were randomized prior to testing to avoid testing bias. Platelet concentrations were determined using quantitative ELISA kits (Quantikine Immunoassays; R&D Systems, Wiesbaden, Germany). All assays have intra-assay co-efficients of variation (CV) of <5% and inter-assay CV of <10%. The lower limits of detection for each individual assay are 10 pg (VEGF) and 5 ng (TSP-1). All tests were performed according to the manufacturer's instructions. All experiments were done at least in duplicate on appropriately diluted samples.

### Microvessel Density (MVD)

Microvessels were stained by using mouse polyclonal anti-CD34 antibody (1∶20 dilution) (Novocastra, Newcastle, UK) as the primary antibody, with binding visualized through the avidin- biotin-peroxidase-complex method. Any red-staining endothelial cell cluster that was clearly separated from adjacent microvessels, tumor cells, and other connective tissue elements was considered a single, countable microvessel. Vessel lumens were not necessary for a structure to be defined as a microvessel. We selected three vascular hotspots both intratumorally and peritumorally for counting CD34 microvessels.

### Statistical Analysis

Statistical analyses were done with SPSS 11.0 (SPSS, Inc, Chicago, IL) software. The data were tested for normality and were found to be non-normally distributed. VEGF, TSP-1 and NPAA distributions in cancer patients and healthy volunteers were compared using the Mann-Whitney *U* test. Correlations were evaluated with the Spearman rank-sum test. The sensitivity and specificity of NPAA were determined by receiver operating characteristic (ROC) curves, and the areas under the curve were calculated [17]. Survival curves were obtained by the Kaplan–Meier method and the Cox proportional hazards model was used to identify factors that were independently associated with survival. The results are presented as median values with interquartile ranges. Significance was presumed at p-values of less than 0.05.

## Results

### NPAA, platelet-derived VEGF and TSP-1 levels in lung cancer patients and healthy controls

NPAA, platelet-derived VEGF and TSP-1 levels were detectable in all healthy controls. Their median values of NPAA, platelet-derived VEGF and TSP-1 levels were 65.4 no. of branch points/10^6^, 21.9 pg/10^6^ and 8.5 ng/10^6^, respectively. Median value of NPAA and VEGF in lung cancer patients were 156.2 no. of branch points/10^6^ (range, 66.4-250.2) and 40.8 pg/10^6^ (range, 15.85–85.9), and significantly higher than that healthy controls (P<0.001 and P = 0.041, respectively; Table 1). There was no significant difference in the platelet-derived TSP-1 levels between lung cancer patients and healthy controls (P = 0.092; Table 1).

**Table 1:** Platelet Derived VEGF, TSP-1Concentration and NPAA in Lung Cancer and Control Study Groups.

Variable	Lung cancer (n = 68)		Healthy controls (n = 68)		*P* value
	Median	Range	Median	Range	
NPAA (no. of branch points/10^6^)	156.2	66.4–250.2	65.4	43.4–123.7	**<0.001**
VEGF(pg/10^6^)	40.8	15.85–85.9	21.9	9.1–62.7	**0.041**
TSP-1(ng/10^6^)	15.4	2.8–58.3	8.5	2.1–21.7	0.092

Bold values indicate “significant difference (*P<*0.05).”

*P* value by Unpaired t-test or Mann–Whitney test.

### Relationship between NPAA, platelet-derived VEGF or TSP-1 levels and clinical pathological profiles

To evaluate the association of NPAA, platelet-derived VEGF or TSP-1 with tumor biology, comparisons of the clinical pathological features with NPAA, platelet-derived VEGF or TSP-1 were made. As expected, patients with high NPAA were more likely to exhibit aggressive clinical pathological features: high NPAA was significantly correlated with poor tumor differentiation (P = 0.004), pathologic stage (P<0.001), pathologic T-factor (P<0.001) and pathologic N-factor (P<0.001), whereas high platelet-derived VEGF was significantly correlated with poor tumor differentiation (P<0.001) and pathologic stage (P<0.001) (Table 2). The levels of platelet-derived TSP were higher in patients in pathologic stage III+IV and pathologic T-factor T2-4 than in patients in pathologic stage I+II (P = 0.002) and pathologic T-factor T1 (P = 0.008), respectively (Table 2). In this study, we found that the median values of NPAA and VEGF in lung cancer patients are significantly higher than that in healthy controls. In contrast, TSP-1 was not elevated in the cancer patients compared with the control samples. Patients with high NPAA were more likely to exhibit aggressive clinical pathological features.

**Table 2:** Platelet Derived VEGF, TSP-1Concentration and NPAA in Lung Cancer.

	No. of patients	NPAA* Median±SD	*P* value	VEGF(pg/10^6^) Median±SD	*P* value	TSP-1(ng/10^6^) Median±SD	*P* value
*Histology*							
Squamous cell carcinoma	28	155.3±66.2	0.47	44.6±17.4	0.09	15.7±8.6	0.31
Non-squamous cell carcinoma	40	143±71.2		44.9±12.2		16.4±7.5	
*Tumor differentiation*							
Poor	32	176.2±75.5	**0.004**	58.6±28.1	**<0.001**	14.8±7.3	0.63
well	36	101.2±49.8		17.8±13.3		15.6±8.1	
*Pathologic stage*							
Stage I + II	38	68.6±42.1	**<0.001**	20.2± 15,4	**<0.001**	15.1±7.9	**0.002**
Stage IIIA, IIIB, or IV	30	159.6±59.7		60.8±23.1		34.5±16.4	
*Pathologic T-factor*							
T1	27	76.4±39.7	**<0.001**	43.7±11.9	0.16	15.1±8.2	**0.008**
T2–4	41	168.3±69.1		39.5±9.8		33.7±19.8	
*Pathologic N-factor*							
N0–1	49	79.3±40.3	**<0.001**	40.4±10.2	0.43	16.3±6.4	0.21
N2–3	19	175.6±74,2		38.9±12.8		14.5±7.2	

Bold values indicate “significant difference (*P*<0.05).”

*P* value by Unpaired t-test or Mann–Whitney test.

*NPAA is expressed in no. of branch points/10^6^

### Receiver-operating curve (ROC) analysis of VEGF and NPAA in the detection of lung cancer

**Figure 1:**
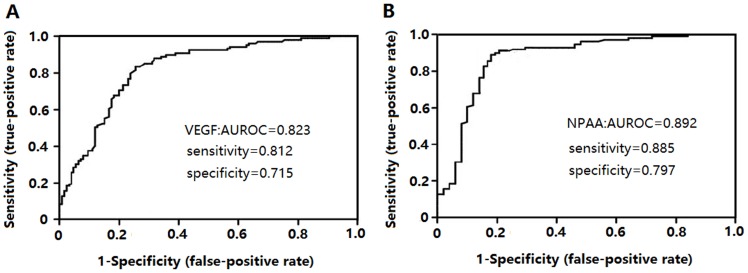
Receiver-operating curve (ROC) analysis of platelet derived VEGF and NPAA in the detection of lung cancer. (A,B) ROC analysis of lung cancer patients and healthy controls, and healthy controls as a negative group and patients with lung cancer as a positive group. NPAA had the best predictive diagnostic accuracy (AUC = 0.893, P<0.0001). AUROC: area under receiver-operating curve.

Figure 1 showed that area under receiver-operating curve (AUROC) of NPAA for differentiating lung cancer from health controls was 0.892 (95% CI, 0.874–0.961), which was higher than 0.823 (95% CI, 0.786–0.941) of VEGF. (P<0.0001).

### Correlations between NPAA, platelet-derived VEGF or TSP-1 levels and patient overall survival or disease-free survival

**Figure 2:**
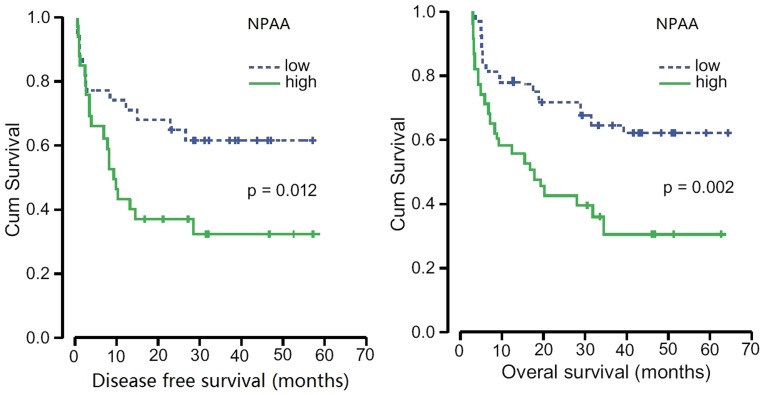
Kaplan-Meier curves of survival differences among NSCLC patients. Disease-free survival (A) and Overall survival (B) for low NPAA and high NPAA patients. P values were determined by the log-rank test.

Univariate Cox regression analysis showed regional lymph node, pathologic stage, NPAA, and platelet-derived VEGF levels to be significant factors affecting overall survival (Table 3). Log-rank analysis showed that elevated NPAA greater than the median were associated with poor disease free survival and poor overall survival (P = 0.012, P = 0.002; Figure. 2), while platelet-derived VEGF and TSP-1 did not display this association. In the case of multivariate analysis for histological differentiation, T factor, regional lymph node, pathologic stage, NPAA, platelet-derived VEGF and TSP-1, only regional lymph node, pathologic stage and NPAA were still related to overall survival in some degree (Table 3). In contrast, regional lymph node, pathologic stage, NPAA were significant factors affecting disease-free survival (Table 4).

**Table 3:** Cox Regression Analysis for Overall Survival in 68 Patients with Lung Cancer.

Variable	Univariate		Multivariate	
	P	HR (95% Cl)	P	HR (95% Cl)
Age	0.143	0.96 (0.93–1.02)	-	-
Histological differentiation	0.186	1.30 (0.88–1.93)	0.208	1.59 (0.77–3.33)
T factor (T1vs T2,3,4)	0.413	1.33 (0.67–2.64)	0.640	0.81 (0.33–1.97)
Regional lymph node (N0,1 vs N2,3)	**0.019**	1.99 (1.10–3.58)	**0.025**	1.95 (1.08–3.52)
Pathologic stage (I, II vs III, IV)	**0.021**	2.13 (1.12–4.04)	**0.024**	1.73 (1.07–2.80)
VEGF (≤Median vs>Median)	**0.037**	1.75 (1.02–2.98)	0.053	1.95 (0.99–5.00)
TSP-1(≤Median vs>Median)	0.088	1.52 (0.91–3.14)	0.289	0.99 (0.63–2.09)
NPAA (≤Median vs>Median)	**0.012**	3.30 (1.30–8.40)	**0.018**	3.04 (1.08–8.55)

Bold values indicate “significant difference (*P<*0.05).”

HR, hazard ratio; Cl, confidence interval.

**Table 4:** Cox Regression Analysis for Disease-free Survival in 68 Patients with Lung Cancer.

Variable	Univariate		Multivariate	
	P	HR (95% Cl)	P	HR (95% Cl)
Age	0.132	0.68 (0.43–1.12)	-	-
Histological differentiation	0.228	1.57 (0.78–2.42)	0.353	1.21 (0.97–2.26)
T factor (T1vs T2,3,4)	0.566	1.83 (0.85–2.81)	0.611	0.91 (0.43–1.77)
Regional lymph node (N0,1 vs N2,3)	**0.030**	1.69 (1.04–2.73)	**0.024**	1.74 (1.08–2.82)
Pathologic stage (I, II vs III, IV)	**0.019**	1.99 (1.12–3.59)	**0.025**	1.93 (1.08–3.30)
VEGF (≤Median vs>Median)	0.052	1.66 (0.94–2.53)	0.073	2.04 (0.86–4.73)
TSP-1(≤Median vs>Median)	0.112	1.62 (0.91–3.76)	0.322	1.29 (0.93–3.27)
NPAA (≤Median vs>Median)	**0.013**	3.52 (1.40–7.61)	**0.008**	2.85 (1.27–7.49)

Bold values indicate “significant difference (*P<*0.05).”

HR, hazard ratio; Cl, confidence interval.

### Relationship between NPAA or Platelet-derived VEGF and Tumor MVD in patients

**Figure 3:**
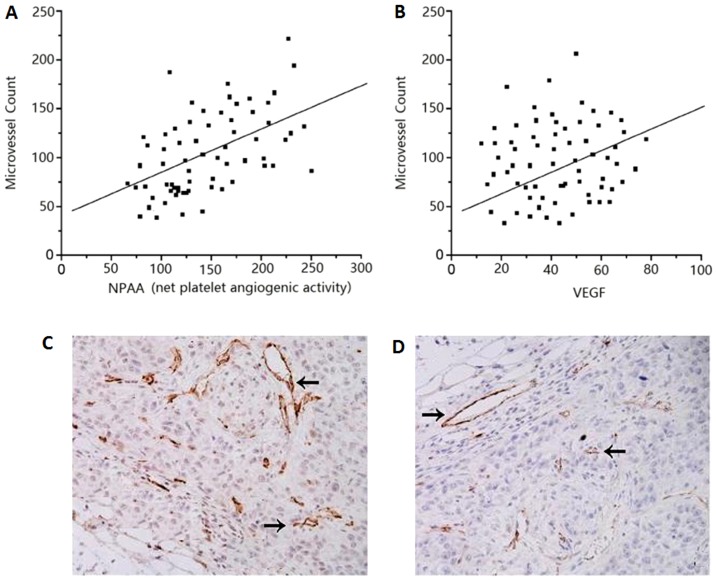
Correlation between tumor MVD and NPAA or platelet-derived VEGF levels. (A) Good correlation between MVD and NPAA in 68 NSCLC patients (r = 0.78; P<0.001). (B) The significant correlation between tumor MVD and platelet-derived VEGF levels (r = 0.521; P<0.01). (C) MVD in NSCLC tissue and (D) normal tissue were determined by CD34 immunohistochemical staining. Arrow, positive stain of MVD.

There was a significant correlation between NPAA or platelet-derived VEGF and tumor MVD in the cancer patients (r = 0.78, P<0.001, r = 0.521; P>0.01; Figure. 3A, B). NPAA have better correlation with tumor MVD than platelet-derived VEGF. These results suggest that NPAA or platelet-derived VEGF may promote tumor angiogenesis. MVD in NSCLC tissue and normal tissue were determined by CD34 immunohistochemical staining (Figure. 3C, D).

### Stimulation of capillary tube formation (CTF) by platelet derived cytokines

**Figure 4:**
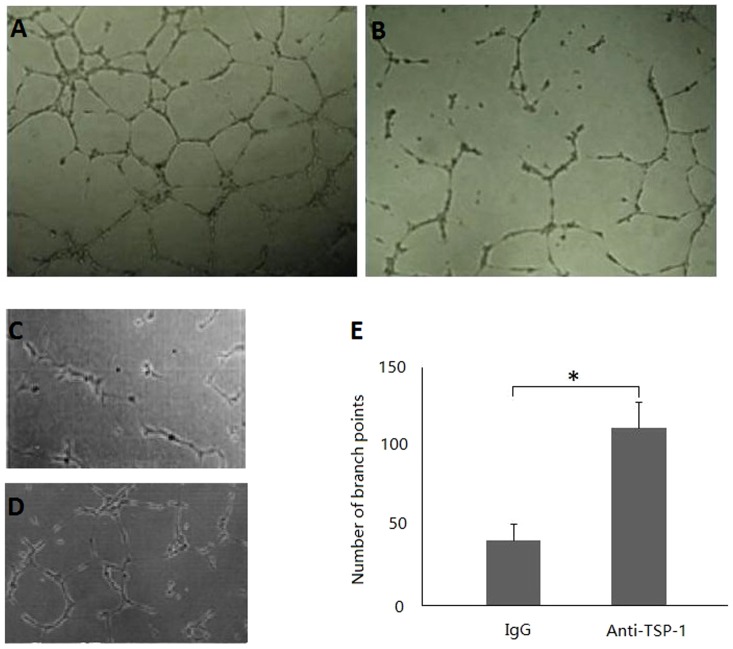
Effect of platelet-released cytokines on angiogenic activity of HUVEC. Representative photographs of the tube formation assay after 18-incubation with platelet-released cytokines are shown in panels A (the patients platelet-released cytokines added) and B (healthy controls platelet-released cytokines added). Preincubation of platelet lysates with IgG control (C) and antiTSP-1 antibody (D). The statistical analysis (E). Bars are means±SD from 4 experiments. * *P<*0.05 control versus treatment.

To evaluate the net platelet angiogenic activity (NPAA), we tested the in vitro effect of platelet lysates from patients with lung cancer and healthy controls on the CTF of HUVECs. The NPAA was significantly higher in the patients than healthy controls (P<0.001;Table 1, Figure. 4A, B).

### The Role of VEGF or TSP-1 in Platelet Lysates from the Patients with NSCLC in NPAA

The preincubation of high-VEGF platelet lysates with antibodies against VEGF resulted in a marked decrease (>48%) in the CTF of HUVEC, whereas only a slight inhibition (<16%) or no inhibition of the CTF of HUVEC was observed when stimulatory platelet lysates with low VEGF levels were preincubated with anti-VEGF antibodies. The preincubation of these inhibitory platelet lysates from cancer patients revealed elevated levels of TSP-1 in both cases with antibodies against TSP-l (200 µg/ml) resulted in an increase in the CTF of HUVEC as compared with these inhibitory platelet lysates preincubated with IgG control (5µg/ml) (*P<*0.05, n = 3) (Figure 4. C,D,E). These findings indicate that intraplatelet TSP-l contributes to the overall anti-angiogenic effect triggered by platelet lysates.

## Discussion

Tumor angiogenesis studies have also shown that circulating platelets are abundant sources of pro-angiogenic molecules for the tumor vasculature, and that tumor cells induce platelet activation and aggregation by the release of platelet-activating factors, resulting in the release of α-granules and their content, which in turn directly affects tumor growth and metastasis [18]–[21]. Elevated platelet counts have been associated with poor prognosis in lung cancer and in gastric cancer, suggesting that a procoagulant state and platelet activation may contribute to early cancer development [22], [23]. It has been reported that VEGF in platelet are elevated and are independent predictors of colorectal carcinoma [24]. Because angiogenesis is regulated by the balance between inhibitors and activators, one aim of our study was to evaluate the TSP-1 (inhibitor)/VEGF (activator) balance in platelets. However, it should be recalled that tumoral angiogenesis is regulated not only by VEGF and TSP-1 levels, but also by a number of identified and unidentified activators and inhibitors that may influence the net angiogenic balance [25], so another aim of our study was to evaluate NPAA.

In this study, we investigated the level of platelet VEGF and NPAA in platelet lysates of 68 patients with NSCLC. We found that the median values of NPAA and VEGF in lung cancer patients are significantly higher than that in healthy controls. Our study further showed that patients with high NPAA are more likely to exhibit aggressive clinical pathological features and that NPAA greater than the median are associated with poor prognosis. The elevated NPAA have better correlation with tumor MVD than platelet-derived VEGF. While elevated platelet counts have been observed in some cancers, we did not observe any significant differences in platelet counts between healthy controls and patients in our population (data not shown).

We measured TSP-1 levels of platelet lysates in NSCLC and found a non-significant elevation in TSP-1 levels. However, the levels of platelet-derived TSP-1 were higher in patients in pathologic stage III+IV and pathologic T-factor T2-4 than in patients in pathologic stage I+II and pathologic T-factor T1. There are two possible explanations for this finding. First, aggressive tumors may generate more TSP-1 than non-aggressive tumors. Secondly, TSP-1 levels may be directly correlated with gross volume of disease, and the patients in this study with aggressive tumors generally had a higher tumor burden. We evaluated the platelet lysates from cancer patients and healthy donors for their ability to increase or decrease CTF of HUVEC as a simple and reproducible *in vitro* approach, gaining information about the NPAA. Indeed, not all platelet samples with high levels of VEGF induced CTF, probably because of the presence of antiangiogenic molecules. Conversely, not all of the stimulatory platelets showed high levels of VEGF, indicating that factors other than VEGF are involved. Whereas tests to identify positive and negative regulators of angiogemesis are cumbersome, our in vitro assay to measure the NPAA of cancer patient on HUVEC CTF provides a simple avenue to functional information on the net balance between angiogenic and anti-angiogenic factors. Such information may be of value in the prognosis of cancer and in monitoring the tumor response to cancer therapy.

In order to further evaluate the clinical value of NPAA, we determined and compared the diagnostic usefulness of NPAA and platelet derived VEGF in cancer and healthy control groups. NPAA levels distinguished lung cancer patients from normal individuals very reliably. NPAA was a significantly better predictor for lung cancer than platelet derived VEGF in this studied population. Survival analysis of the lung cancer patients was evaluated in this study based on the levels of NPAA. In our study, survival rate of patients with high NPAA was significantly lower than the patients with low NPAA. Of note, preoperative levels of platelet derived VEGF in these patients did not correlate with a poor outcome (data not shown).To our knowledge, this is the first report concerning NPAA in NSCLC.

There are multiple mechanisms by which the various angiogenesis regulatory proteins could become increased in the platelets of cancer patients. First, platelets could directly acquire a factor from the tumor via endocytosis. Second, the tumor could stimulate the megakaryocyte to upregulate the synthesis of an angiogenesis regulatory protein. Third, because platelets contain mRNAs and translational machinery, platelets could also be stimulated to synthesize an angiogenesis regulatory protein [24].

In conclusion, the results of this study showed that while NPAA, platelet derived VEGF and TSP-1 levels were higher in NSCLC patients before surgery than in healthy controls, only both NPAA and platelet derived VEGF levels showed a statistically significant difference between the two groups. It also showed that NPAA greater than the median were associated poor prognosis. Although platelet derived TSP-1 level appears not to be a marker for NSCLC, NPAA may be more of value than platelet derived VEGF in the diagnostic and prognosis of non-small cell lung cancer.

## References

[1] Zhang SW, Chen WQ, Kong LZ (2007) An annual report: cancer incidence in 35 Cancer Registries in China, 2003. Bullet. Chin Cancer 16: 494–507.

[2] Parkin DM, Bray F, Ferlay J (2005) Global cancer statistics, 2002. CA Cancer J Clin 55: 74–108. 15761078 10.3322/canjclin.55.2.74

[3] Youlden DR, Cramb SM, Baade PD (2008) The international epidemiology of lung cancer: geographical distribution and secular trends. J Thoracic Oncol 3: 819–821. 10.1097/JTO.0b013e31818020eb 18670299

[4] Folkman J, Browder T, Palmblad J (2001) Angiogenesis research: guidelines for translation to clinical application. Thromb Haemost 86: 23–33. 11487011

[5] Kaya M, Wada T, Akatsuka T, Kawaguchi S, Nagoya S, et al (2000) Vascular endothelial growth factor expression in untreated osteosarcoma is predictive of pulmonary metastasis and poor prognosis. Clin Cancer Res 6: 572–577. 10690541

[6] Takeda A, Shimada H, Imaseki H, Okazumi S, Natsume T, et al (2000) Clinical significance of serum vascular endothelial growth factor in colorectal cancer patients: correlation with clinicopathological factors and tumor markers. Oncol Rep 7: 333–338. 10671682

[7] Fontanini G, Vignati S, Boldrini L, Chinè S, Silvestri V, et al (1997) Vascular endothelial growth factor is associated with neovascularization and influences progression of non-small cell lung carcinoma. Clin Cancer Res 3: 861–865. 9815760

[8] O‘Byrne KJ, Koukourakis MI, Giatromanolaki A, Cox G, Turley H, et al (2000) Vascular endothelial growth factor, platelet-derived endothelial cell growth factor and angiogenesis in non-small-cell lung cancer. Br J Cancer 82: 1427–32. 10780522 10.1054/bjoc.1999.1129 PMC2363365

[9] Good DJ, Polverini PJ, Rastinejad F, Mikkelsen T, Polverini PJ, et al (1990) A tumor suppressor-dependent inhibitor of angiogenesis is immunologically and functionally indistinguishable from a fragment of thrombospondin. Proc Natl Acad Sci USA 87: 6624–6628. 1697685 10.1073/pnas.87.17.6624 PMC54589

[10] Taraboletti G, Roberts D, Liotta LA (1990) Giavazzi (1990) Platelet thrombospondin modulates endothelial cell adhesion, motility, and growth: a potential angiogenesis regulatory factor. J Cell Biol 111: 765–72. 1696271 10.1083/jcb.111.2.765 PMC2116188

[11] de Fraipont F, Nicholson AC, Feige JJ, Van Meir EG (2001) Thrombospondins and tumor angiogenesis. Trends Mol Med 7: 401–407. 11530335 10.1016/s1471-4914(01)02102-5

[12] Tuszynski GP, Smith M, Rothman VL, Capuzzi DM, Joseph RR, et al (1992) Thrombospondin levels in patients with malignancy. Thromb Haemost 67: 607–611. 1509400

[13] Nathan FE, Hernandez E, Dunton CJ, Treat J, Switalska HI, et al (1994) Plasma thrombospondin levels in patients with gynecologic malignancies. Cancer 73: 2853–2858. 8194026 10.1002/1097-0142(19940601)73:11<2853::aid-cncr2820731131>3.0.co;2-9

[14] Lindemann S, Tolley ND, Dixon DA, McIntyre TM, Prescott SM, et al.. (2001) Activated platelets mediate inflammatory signaling by regulated interleukin 1beta synthesis. J Cell Biol 154:485– 90. 10.1083/jcb.200105058 PMC2196422 11489912

[15] Larrivee B, Karsan A (2005) Isolation and culture of primary endothelial cells. Methods Mol Biol 290: 315–329. 15361671 10.1385/1-59259-838-2:315

[16] World Health Organization (1982) Histological typing of lung tumors. Am J Clin Pathol 77: 123–136. 7064914 10.1093/ajcp/77.2.123

[17] Pepe MS (2003) The statistical evaluation of medical tests for classification and prediction. Oxford University Press, New York, pp 66–95.

[18] Gonzalez FJ, Rueda A, Sevilla I, Alonso L, Villarreal V, et al (2004) Shift in the balance between circulating thrombospondin-1 and vascular endothelial growth factor in cancer patients: relationship to platelet alpha-granule content and primary activation. Int J Biol Markers 19: 221–228. 10.5301/JBM.2008.1959 28207087

[19] Verheul HMW, Hoekman K, Lupu F, Broxterman HJ, van der Valk P, et al (2000) Platelet and coagulation activation with vascular endothelial growth factor generation in soft tissue sarcomas. Clin Cancer Res 6: 166–171. 10656446

[20] Bastida E, Ordinas A (1988) Platelet contribution to the formation of metastatic foci: the role of cancer cell-induced platelet activation. Haemostasis 18: 29–36. 3047021 10.1159/000215780

[21] Nierodzik ML, Karpatkin S (2006) Thrombin induces tumor growth, metastasis, and angiogenesis: evidence for a thrombin-regulated dormant tumor phenotype. Cancer Cell 10: 355–362. 17097558 10.1016/j.ccr.2006.10.002

[22] Pedersen LM, Milman N (1996) Prognostic significance of thrombocytosis in patients with primary lung cancer. Eur Respir J 9: 1826–830. 8880098 10.1183/09031936.96.09091826

[23] Ikeda M, Furukawa H, Imamura H, Shimizu J, Ishida H, et al (2002) Poor prognosis associated with thrombocytosis in patients with gastric cancer. Ann Surg Oncol 9: 287–291. 11923136 10.1007/BF02573067

[24] Peterson JE, Zurakowski D, Italiano JE Jr, Michel LV, Connors S, et al (2012) VEGF, PF4 and PDGF are elevated in platelets of colorectal cancer patients. Angiogenesis 15: 265–273. 22402885 10.1007/s10456-012-9259-z

[25] Hanahan D, Folkman J (1996) Patterns and emerging mechanisms of the angiogenic switch during tumorigenesis. Cell 86: 353–364. 8756718 10.1016/s0092-8674(00)80108-7

